# Effect of gallium environment on infrared emission in Er^3+^-doped gallium– antimony– sulfur glasses

**DOI:** 10.1038/srep41168

**Published:** 2017-01-20

**Authors:** Qing Jiao, Ge Li, Lini Li, Changgui Lin, Guoxiang Wang, Zijun Liu, Shixun Dai, Tiefeng Xu, Qinyuan Zhang

**Affiliations:** 1 Laboratory of Infrared Materials and Devices, The Research Institute of Advanced Technologies, Ningbo University, Ningbo 315211, China; 2 Key Laboratory of Photoelectric Detection Materials and Devices of Zhejiang Province, Ningbo, 315211, China; 3 State Key Laboratory of Luminescence Materials and Devices, South China University of Technology, Guangzhou 510641, China

## Abstract

Gallium-based Ga–Sb–S sulfide glasses was elaborated and studied. A relationship between the structure, composition, and optical properties of the glass has been established. The effects of the introduction of Ga on the structure using infrared and Raman spectroscopies and on the Er^3+^-doped IR emission have been discussed. The results show that incorporation of Ga induced the dissociation of [SbS_3_] pyramids units and the formation of tetrahedral [GaS_4_] units. The dissolved rare earth ions are separated around the Ga–S bonding and the infrared emission quenching are controlled. Moreover, continuous introduction of Er ions into the glass forms more Er–S bonds through the further aggregation surrounding the [GaS_4_] units. In return, the infrared emission intensity decreased with excessive Er ion addition. This phenomenon is correlated with the recurrence concentration quenching effect induced by the increase of [GaS_4_] units.

Chalcogenide glasses doped with rare earth (RE) ions attracted considerable attention due to their applications in medical surgery, military countermeasures, atmosphere pollution monitoring, remote sensing, and eye-safe laser radar^1^,^2^,^3^,^4^. The fiber lasers operating at mid-infrared (MIR) wavelength can be obtained from various RE lasing ions, such as Dy^3+^, Ho^3+^, Pr^3+^, Tm^3+^, and Er^3+^ 5,6,7. Among them, Er-doped glasses are attractive for use in integrated optoelectronics because of the Er^3+^ intra-4f emissions at the standard telecommunications wavelength at 1540 nm^8^,^9^,^10^. Furthermore, Er^3+^-doped system presents the laser emission at about 2.7 μm, which is close to the most pronounced absorption band of water at 3 μm. To obtain high luminescence efficiency, glass with low phonon energy is required as matrix for Er^3+^ ions doping to decrease the multi-phonon relaxation rate. Chalcogenide glasses have been considered as promising materials for MIR emission lasers due to the very low phonon energies (150–450 cm^−1^) and high stimulated emission cross sections^11^,^12^. However, RE ions are not easily introduced into the host with high coordination number because chalcogenide glasses are mostly covalent solids with rigid networks and unable to satisfy the coordination environment^13^. Therefore, the issue of the limited solubility induced low fluorescence efficiency is still a great challenge. Recently, many studies indicated that the incorporation of gallium as modifiers into chalcogenide glassy networks could dramatically increase the solubility of RE ions due to the presence of edge-sharing [GaS_4_] tetrahedral structure^14^,^15^,^16^. The incorporation of Ga provides a compensation for the negative charge of free S^2−^ ions by forming the chemical bonds with RE ions. The expecting compositions, including Ga–La–S^17^, Ga–As–S^18^, Ga–Na–S^19^, and Ga–Ge–S glasses^20^ *et al*. have been developed as host materials for RE dopants because a large amount of RE ions could be dissolved and dispersed in low phonon energy hosts homogeneously, making the doped glasses be promising for IR amplifiers or lasers.

Recent studies indicated that Ga-based Ga_2_S_3_–Sb_2_S_3_ glass systems not only possess good thermal ability but also exhibit higher non-linear susceptibility and Raman gain coefficients, also including the RE (Dy^3+^/Tm^3+^)-doped infrared emissions^21^,^22^,^23^,^24^. In the current work, the Ga composition was tuned in the Sb–S glassy phosphors to find out the trade-off between glass structure and achieving efficient MIR emissions. To obtain the structural information about the local environment in these glasses, far-infrared absorption measurement was performed. Structural information was also obtained by Raman analysis and high resolution transmission electron microscope (HRTEM) as a function of different Ga concentration. In addition, Er^3+^ ions infrared emission induced by structure evolution was investigated hoping to explore the way of improve RE infrared emission in chalcogenide glass systems.

## Results and Discussion

**Figure 1:**
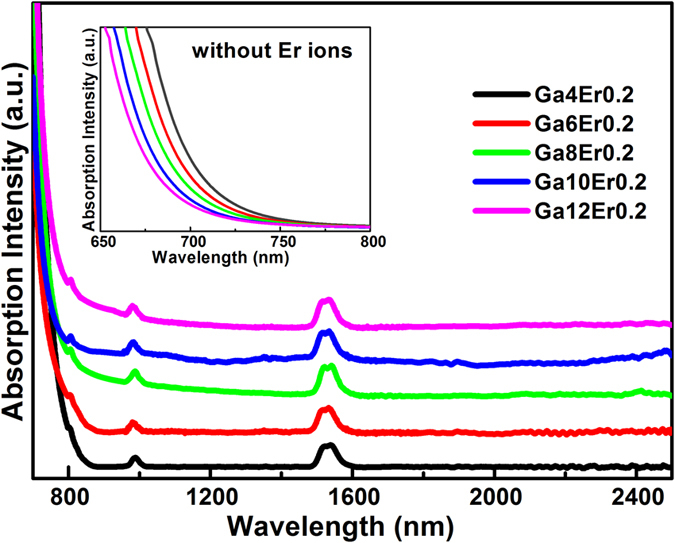
Absorption spectrum of Er^3+^-doped glass samples as a function of Ga concentrations.

Figure 1 shows the absorption spectra of Er^3+^-doped Ga–Sb–S (GSS) glasses in the range of 400–2500 nm at room temperature and the absorption bands of Er^3+^ corresponding to the transitions labeled starting from the ground state to the higher levels. The electronic intra-4f transitions from the ground energy level ^4^I_15/2_ to the higher energy state ^2S+ 1^L_*J*_ are located at 1540 nm (^4^I_15/2_ → ^4^I_13/2_), 990 nm (^4^I_15/2_-^4^I_11/2_), and 810 nm (^4^I_15/2_-^4^I_9/2_), respectively. The results indicate that the doped Er^3+^ ions are successfully introduced into the glass matrix. The obvious absorption around 980 nm suggests that this glass can be pumped efficiently with a 980 nm laser diode. In the inset of the undoped GGS glasses, one can see that a shift of absorption edge to the short wavelength occurred with increase of Ga concentration, which means the microstructure change inside the glass network.

**Figure 2:**
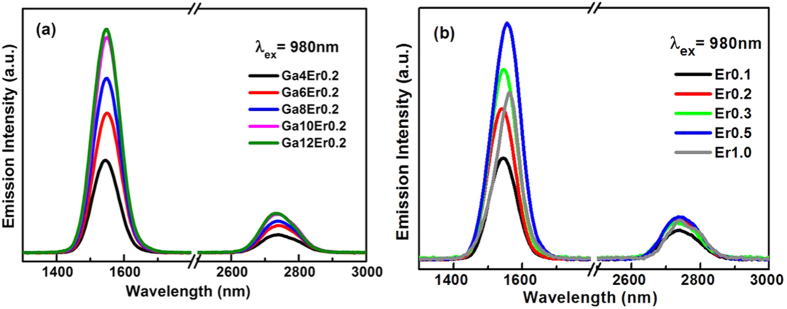
Infrared emission spectrum of Er^3+^-doped samples under 980 nm laser excitation (**a**) with increase of Ga concentration and (**b**) Er ions concentration.

The infrared emission spectra of Er^3+^-doped glass samples are demonstrated ranging from 1000 nm to 3000 nm under 980 nm light excitation in Fig. 2. Two significant emission bands centered at 1.5 and 2.7 μm are attributed to the transitions ^4^I_13/2_ → ^4^I_15/2_ and ^4^I_11/2_ → ^4^I_13/2_, respectively. Moreover, the emission intensity at 1.5 and 2.7 μm increased with addition of gallium component as shown in Fig. 2(a), in which the maximum emission was obtained in sample containing the most Ga component. The results implied that the ability of the Er^3+^ radiative transition was promoted with gallium element by inducing a structured environment. According to the reports, the solubility of RE ions can be significantly improved by adding gallium in the chalcogenide glass matrix^15^,^25^,^26^. Ga was proposed to be associated with the formation of [GaS_4_] tetrahedra in the vitreous network^27^,^28^,^29^. The increased [GaS_4_] tetrahedra along with introduction of Ga element may have provided the optimization of the energy transition environment around RE ions, such as the chemical bonds between Ga and RE ions, which compensate for the negative charge of free S^2−^ ions. Most results focused on the increased solubility of RE ions in the presence of gallium since the first report of the incorporation of Ga in As_2_S_3_ glasses by Kolomiets *et al*. in 1960^30^. We need to ensure that the effect of Ga addition induced structure linkage on the RE emissions in glasses.

Figure 2(b) shows the infrared emissions in Ga8 glass sample as a function of Er^3+^ concentration. Notable maximum emission intensity at 1.5 and 2.7 μm was obtained until 0.5 mol% Er^3+^ was introduced into the glass matrix. The concentration quenching effect is observed in 1.0 mol% RE-doped sample. Comparing with Tm^3+^ doped sol-gel glass, the optimum concentration of Tm^3+^ was improved to 0.8 mol%, owing to the La/Al co-doping as network modifiers to decrease the clustering quenching effect of RE ions^31^. As glass modifiers, La, Ga, Al, Y and P were usually used to optimize the microstructure environment around RE ions in glass materials. In this work, Ga was introduced as a modifier to improve the solubility of RE ions in GSS glasses. The RE ions seemed to be surrounded by 6 or 7 sulfur atoms in chalcogenide glassy matrix^29^,^32^,^33^ and incorporated on sites close to the gallium atoms to balance the partial negative charge of tetrahedral [GaS_4_] units^34^. The introduction of Ga into Ge–As–S glasses greatly enhanced RE solubility and dispersal, particularly for Ga:RE ratios ≥ 10:1^35^. The peak position exhibited an obvious red-shift in the 1 mol% Er^3+^-doped glass sample, which coincide with the ratio exceeding the critical point. As reported by Petr Nemec through mass spectra measurement, this phenomenon was attributed to the fact that the two erbium-containing species contain gallium atoms (GaSb_2_SEr^+^ and GaS_6_Er^2+^)^36^. The second cluster corresponds to the structural fragment (suggested in the literature for high concentration of erbium and gallium), which could be involved in the clustering effect of the higher addition of Ga and Er samples. Therefore, the RE site transformation was the possible reason for the emission performance.

**Figure 3:**
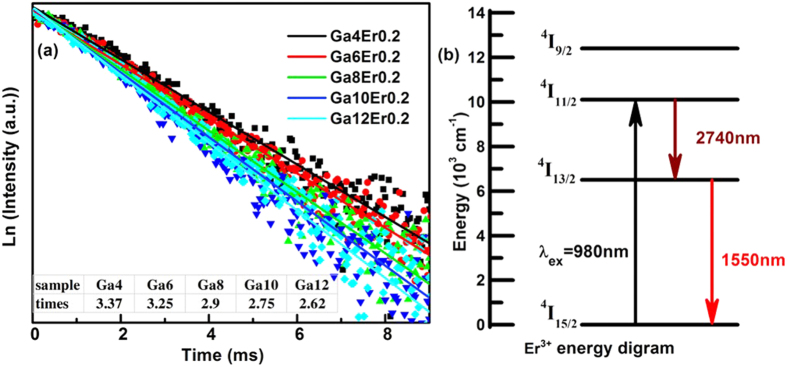
(**a**) The fluorescence decay curves of Er^3+^: ^4^I_13/2_ level in Er^3+^-doped GSS glasses with variation of Ga concentration at 980 nm. (**b**) The energy transfer sketch of Er^3+^-doped glasses when pumped at 980 nm.

Figure 3(a) shows the decay curves of Er^3+^:1.5 μm emission in GSS glasses at room temperature. The lifetime of Er^3+^:^4^I_13/2_ level was shortened from 3.37 ms to 2.75 ms with increase of Ga into the glass host. According to the radiative transition theory of Einstein, the radiative transition probability is proportional to the reciprocal of fluorescent lifetime, which is 297 sec^−1^, 308 sec^−1^, 345 sec^−1^, 364 sec^−1^ and 382 sec^−1^ respectively with introduction of Ga component in this work. Comparing with Dy^3+^ doped glass, the lifetimes obtained by the single exponent fitting method are 3.62 ms and 1.42 ms, which is measured at 2.95 and 4.40 μm. The radiative transition probability is calculated as 276 and 704 sec^−1^, respectively^37^. While in Tm^3+^ doped present GaSbS glass, the radiative transition probability of emission at 3.8 μm is about 500 sec^−1 ^21. It shows that different rare earth ions in the same glass system presented on a different radiative transition probability. In Er^3+^-doped GeGaSbS glasses, the radiative transition probability corresponding to 1500 nm is about 714 and 588 sec^−1^ when the dopant concentration is 500 ppm and 10000 ppm, respectively^38^. The comparative results further showed that the radiative transition probability of rare earth ions is influenced by multiple factors. The result identified that the Ga-based environment promoted the energy transition of doped RE^3+^ ions, therefore leading to the increase of the radiative transition probability of electrons on the upper energy. The more electrons were expensed for generation of infrared emission photos, the stronger emission intensity was obtained. Similarly, the emission at 2.7 μm was increased simultaneously, although the relative increase was much lower. As can be explained by the energy diagram in Fig. 3(b), the 1.5 μm energy transition efficiency was higher than that of 2.7 μm emission. The reason was that 2.7 μm emission belongs to the energy transition between the excitation states, which need much lower phonon energy. The non-radiative transition with phonon assisted participated in the lower excitation energy transition, thus generating more 1.5 μm emissions. The phonon energy is a key factor for the mid and far infrared emissions in chalcogenide glasses, which is determined by the glass compositions and the type of network connections. Therefore, we need to ensure the structure units of the glass host, which is always a research spot and a problem.

**Figure 4:**
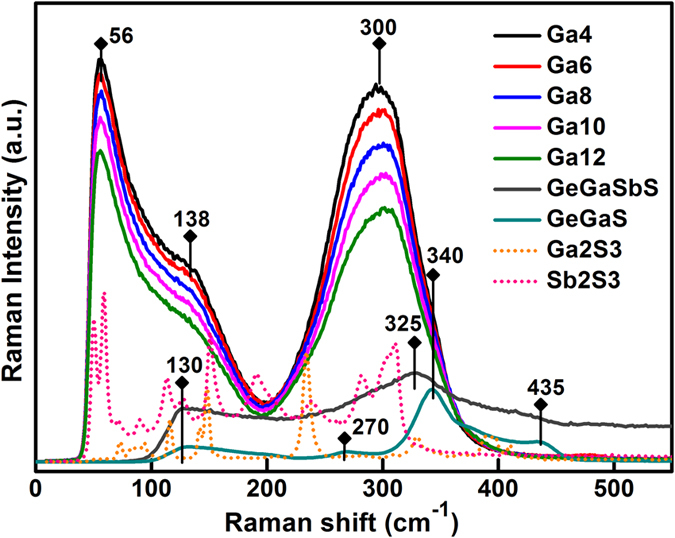
Raman spectra of GSS host glasses with different Ga concentrations and the corresponding reference samples including Ga_2_S_3_, Sb_2_S_3_ crystals, and GeGaS, GeGaSbS glasses.

To explore the structure details of gallium-based samples of antimony sulfide matrix, the Raman spectra is displayed in Fig. 4. For comparison, the vibration spectra of Ga_2_S_3_, Sb_2_S_3_ crystals, and GeGaS, GeGaSbS glasses were added as a reference in the picture. Three vibration bands were dominated in GSS glasses, including peak at ~56 cm^−1^ and broad bands centering at ~138 and ~300 cm^−1^. The origin of the band in the lower frequency at ~56 cm^−1^ is not totally clear. Compared with the Raman spectra of Sb_2_S_3_ crystals and two referenced glasses, this band may be associated with the vibrations of Sb–S bonds because no similar vibrations band are detected in the GeGaS and GeGaSbS glasses where the characteristic peak is located in the Sb_2_S_3_ crystal. In accordance with the distinct vibrations of XY_4_ tetrahedral molecules in ref. 39 and the reported ~130 cm^−1^ vibration corresponding to [GeS_4_] tetrahedra in GeGaS and GeGaSbS glasses, the broad Raman band peaking at ~138 cm^−1^ can be attributed to the symmetrical bending (V_2_) and asymmetrical bending (V_4_) vibration of [GaS_4_] units^40^. Owing to the transformation of mode V_2_ to V_4_, the amplitude of this band is possibly broadened which is consistent with the increasing quantity of [GaS_4_] units. There are reports indicated that pyramid units [SbS_3_] and tetrahedron structure [GaS_4_] are vibrated in the ~290 and ~340 cm^−1^ bands^41^,^42^, with the vibrations of metal–metal bonds of S_3_Ga–GaS_3_ type at ~265 cm^−1 ^41,43. Therefore, the peak at ~300 cm^−1^ was attributed to the superimposed bands of [GaS_4_] and [SbS_3_] units. Moreover, the strong broad band in the range of 230–350 cm^−1^ presented a slight shift to the higher frequency, which implies that the [GaS_4_] structure units increased in the host. The results preliminarily confirmed that the molecular structure of our GSS glass matrix consists of [SbS_3_] pyramids and [GaS_4_] tetrahedra randomly linked by shared sulfur atoms surrounded with part of the Ga–Ga related metal–metal bonds. Addition of Ga_2_S_3_ into the glasses resulted in the micro changes of network connections and vibration modes of structure units. This demonstrated that [GaS_4_] units possibly contributed to the infrared emission of doped RE ions.

**Figure 5:**
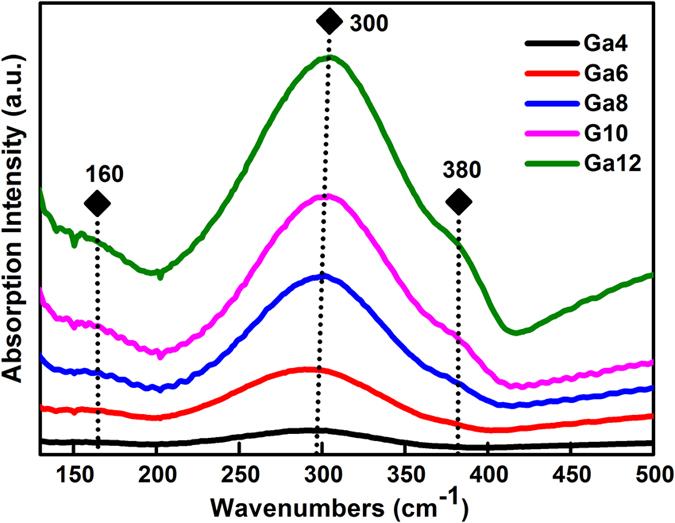
Far infrared absorption spectra of Ga component varied GSS glass samples.

**Figure 6:**
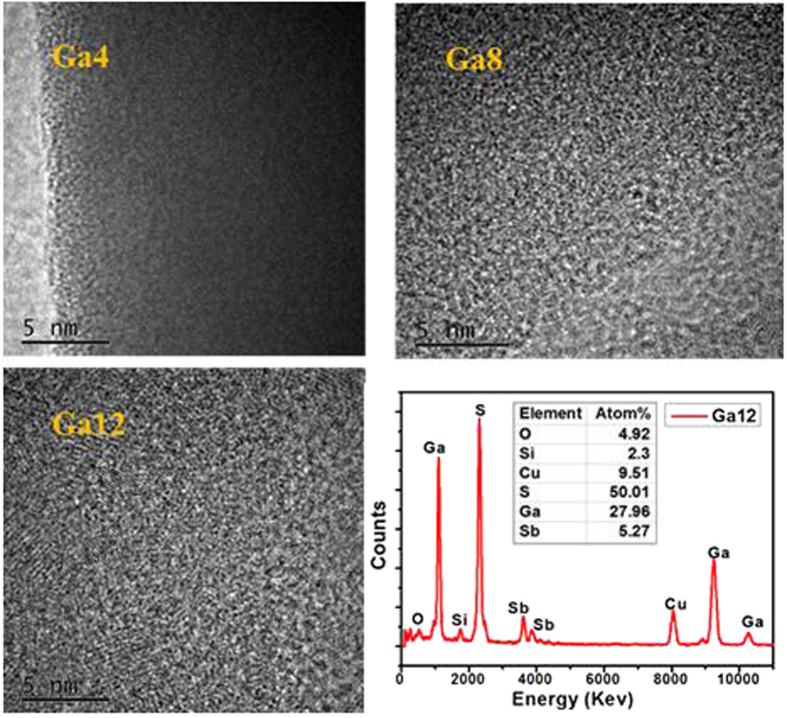
The HR-TEM image of Ga4, Ga8 and Ga12 glass samples and the corresponding EDS spectra of the crystalline area in Ga12 sample. The inset is related quantity of atomic percentage.

Figure 5 shows the far infrared absorption spectra of Ga-containing glass samples ranging from 100 cm^−1^ to 450 cm^−1^ for further analysis of the coordination environment around RE ions. Two vibration bands are positioned in ~160 and ~300 cm^−1^, which is mainly due to the vibration of the Sb–S band. As reported in S. Bamier *et al*.’s research, the Sb–S band mainly existed in the form of [SbS_3_] pyramid structure unit with sulfur atoms located at the vertices^22^. Moreover, the presence of the two bands in the range of ~270–290 and ~300–335 cm^−1^ were attributed to the V_3_ and V_1_ mode of [SbS_3_], respectively, based on the molecular model proposed by Lucovsky and Matrin for the vibrational spectra of chalcogenide glasses^40^,^44^. The shift of the broad band to the higher wavenumber indicated the mode transformation of V_1_ to V_3_ of [SbS_3_] pyramids with introduction of Ga element. The introduction of Ga kept the basic framework with no change of glass structure in the short range. When the proportion of the Ga component attained 10 mol%, another wide vibration band appeared in the range of 330–375 cm^−1^. This extra band was attributed to the vibration of V_3_ and V_4_ modes belonging to [GaS_4_] tetrahedron^22^. Herein, the similar wide band was significantly detected in the sample containing 12 mol% Ga, which is obviously dominated in the 380 cm^−1^. Thus, apparent vibration of [GaS_4_] units is present. And it was also identified with high resolution transmission electron microscopy (HRTEM) image of Ga4, Ga8 and Ga12 samples in Fig. 6. In the lower Ga4 contained sample, it exhibited uniform amorphous state in the whole glass. With increase of Ga component, it presented on an aggregation phenomenon, meaning the gradual formation of [GaS_4_] units in in Ga8 sample. When the concentration of Ga attained at 12 mol%, barely at the edge of the glass forming range, the clustering effect becomes evidently and evenly distributed in the glass host with some crystal lines observed in the HRTEM picture. The corresponding EDS result of the Ga12 sample is also listed. It is evident that Ga and S peaks are clearly observed in the crystalline area and their atomic proportion is up to 78% meaning the most [GaS_4_] units are formed. The small amount of O and Si peaks belongs to the quartz tube used in the preparation process. And the component of Sb is a little observed which is belong to the component of the matrix. The results suggested that large amount of [GaS_4_] units formed and evenly distributed with increase of Ga addition in the glass host, which is well in accordance with Raman and FTIR results.

**Figure 7:**
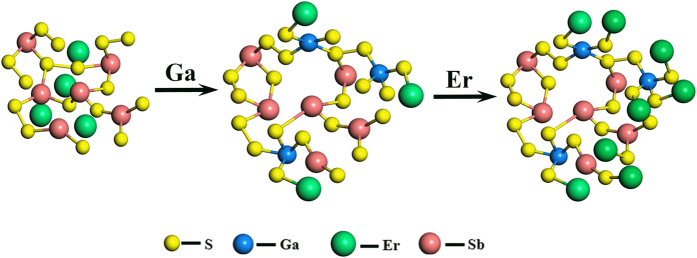
Schematic illustration of structure evolution with increasing of Ga and Er^3+^ ions concentration in host glasses.

With the above information in mind and on the basis of the results, the structure of GSS glasses can be visualized as follows. The basic structural units forming the backbone of the network structure are [SbS_3_] pyramids and [GaS_4_] tetrahedra connected through a bridging sulfur. Little fraction of metal–metal bonds appeared, which are most probably Ga–Ga with increasing Ga_2_S_3_ concentration. Simultaneously, the vibration strengthen of [GaS_4_] units was improved and the vibration mode developed from V_2_ to V_4_ indicating the systematic to asymmetrical bending vibrations. Not only that, variation of pyramid [SbS_3_] vibration was observed in the V_3_ asymmetric stretching and V_1_ symmetric stretching mode with addition of Ga_2_S_3_ proportion. The vibration mode changes implied the alteration of the microstructure inside the glass host. The details of the structure bonding transformation with addition of Ga element are depicted in Fig. 7.

In the lower Ga-containing sample, the glass structure was predominated by the Sb–S linkage framed [SbS_3_] pyramid three-dimensional network. The doped RE^3+^ ions are dispersed in the Sb–S covalent network, tending to aggregate together owing to the mismatch coordination environment. The network directly induced the limited RE solubility and the fast fluorescence quenched effect. Interestingly, with addition of Ga component, the framework of the [SbS_3_] units was broken and bonded with [GaS_4_] tetrahedron. Owing to the negative charge of the [GaS_4_] units, the doped RE^3+^ ions connected with the tetrahedron structure to balance the electric neutrality and the matrix stability. Therefore, RE^3+^ ions were dispersed evenly, and the quenching effect was further prevented. The corresponding infrared emission was then improved with addition of Ga element. After more Er^3+^ ions were doped in the matrix, the obvious red shift of the peak position in the emission spectra was observed. The reason is that the network bonding transformation, such as GaSb_2_SEr^+^ to GaS_6_Er^2+^, occurred when more Er^3+^ ions connected with [GaS_4_] units, in which the latter cluster is inclined to generate concentration quenching. Thus, the balanced amount of glass modifier mechanism, including the effect of the group vibration mode changes, needs to be established for the optimization of the infrared emissions in chalcogenide glasses.

In summary, chalcogenide glasses of Ga–Sb–S systems were successfully prepared, and the effect of Ga addition on the glass structure evolution and doped RE^3+^ ions emission was better modified. The FTIR and Raman results demonstrated that the rare earth first dispersed in the Sb–S bonding [SbS_3_] pyramid units consists of glass network. With increase of Ga concentration, [GaS_4_] tetrahedron units were connected with Er ions, leading to the decrease of the aggregation effect. The radiative transition probability of the rare earth ions was raised efficiently. The Ga atoms induced network arrangement is a potential way to obtain the enhancement of infrared quantum efficiency in RE-doped chalcogenide glasses.

## Methods

### Synthesis of glass materials

Chalcogenide glasses of Ga_*x*_Sb_(40−*x*)_S_60_: Er_*y*_ (GSS), where *x* = 4, 6, 8, 10, 12 mol% and *y* = 0, 0.1, 0.2, 0.3, 0.5, 1.0 mol% were synthesized by a standard melt-quenching technique from high pure elements of Ga (5N), Sb (5N), S (5N), and Er_2_S_3_ (5N). The samples were named as Ga4, Ga6, Ga8, Ga10 and Ga12 dependent of Ga concentrations. The glass forming ability decreased a lot when more Ga component such as 15 mol% was employed. The elements were weighted into silica glassy ampoules in a glove box with dry argon atmosphere, evacuated to residual pressure of ~10^−3^ Pa, sealed before the ampoule was placed into a rocking furnace, and heated at 980 °С for 12 h. The melts of the glasses were quenched into water, and the glasses were annealed at T = T_g_ − 20 K for 5 h to relax the mechanical strain. The furnace was switched off after this time, and the samples were left there for slow cooling to room temperature. The bulk glasses were cut into discs with parallel faces, diameter of ~9 mm, and thickness of ~1.5 mm. The discs were both polished into rectangular shape with submicrometer-sized diamond particles to the optical quality.

### Characterization of samples

The optical absorption spectra were recorded by using a double-beam UV-Vis-NIR spectrometer (JASCO 570) in the spectral range of 350–2500 nm to find 4f–4 f electronic transitions of Er^3+^ ions as well as the fundamental optical absorption of the glassy host matrix. Experiments of FTIR absorption spectra were carried out in the 100–450 cm^−1^ range by using an Equinox55 spectrometer equipped with a KBr detector. Photoluminescence was measured in the spectral laser pumped with diodes lasers operating at wavelength of 980 nm with a maximum power of 2 W as excitation source. The near- and mid-infrared fluorescence emission spectra were measured with FLS 980 (Edinburg Co., England) and detected with a liquid-nitrogen-cooled PbS detector. The fluorescence decaying curve was recorded with a digital oscilloscope. The Raman spectrum was measured by a Renishaw Micro-Raman instrument. A 300 kV field emission analytical transmission electron microscope (HRTEM, FEI tecnai G2F30) equipped with an energy dispersive X-ray spectroscope (Oxford X-MAX) was used to observe the structure clustering phenomenon and made element analysis. All the measurements were carried out at room temperature.

## Additional Information

**How to cite this article**: Jiao, Q. *et al*. Effect of gallium environment on infrared emission in Er^3+^-doped gallium– antimony– sulfur glasses. *Sci. Rep.* **7**, 41168; doi: 10.1038/srep41168 (2017).

**Publisher's note:** Springer Nature remains neutral with regard to jurisdictional claims in published maps and institutional affiliations.

## References

[b1] Eggleton B. J., Luther-Davies B. & Richardson K. Chalcogenide photonics. Nat. Photon. 5, 141–148 (2011).

[b2] Zakery A. & Elliott S. R. Optical properties and applications of chalcogenide glasses: a review. J. Non-Cryst. Solids 330, 1–12 (2003).

[b3] Han Z. . On-chip mid-infrared gas detection using chalcogenide glass waveguide. Appl. Phys. Lett. 108, 201–210 (2016).

[b4] Demetriou G. . Refractive index and dispersion control of ultrafast laser inscribed waveguides in gallium lanthanum sulphide for near and mid-infrared applications. Opt. Express 24, 6350–6358 (2016). 27136826 10.1364/OE.24.006350

[b5] Tian Y., Xu R., Hu L. & Zhang J. Intense 2.7 μm and broadband 2.0 μm emission from diode-pumped Er^3+^/Tm^3+^/Ho^3+^-doped fluorophosphate glass. Opt. Lett. 36, 3218–3220 (2011). 21847213 10.1364/OL.36.003218

[b6] Yang S. . Broadband near-infrared emission in Tm^3+^-Dy^3+^ codoped amorphous chalcohalide films fabricated by pulsed laser deposition. Opt. Express 19, 26529–26535 (2011). 22274237 10.1364/OE.19.026529

[b7] Karaksina E. V. . Preparation of high-purity Pr^3+^ doped Ge-As-Se-In-I glasses for active mid-infrared optics. J. Lumin. 177, 275–279 (2016).

[b8] Polman A. Erbium implanted thin film photonic materials. J. Appl. Phys. 82, 1–39 (1997).

[b9] Ivanova Z. G. . Photoluminescence of Er^3+^ ions in (GeS_2_)_80_(Ga_2_S_3_)_20_ glasses. J. Optoelectron. Adv. Mater. 7, 349–352 (2005).

[b10] Camargo A. S. S. D. . 2.8 and 1.55 μm Emission from Diode-Pumped Er^3+^-doped and Yb^3+^ Codoped Lead Lanthanum Zirconate Titanate Transparent Ferroelectric Ceramic. Appl. Phys. Lett. 86, 241112–241113 (2005).

[b11] Seddon A. B., Tang Z., Furniss D., Sujecki S. & Benson T. M. Progress in rare-earth-doped mid-infrared fiber lasers. Opt. Express 18, 26704–26719 (2010). 21165021 10.1364/OE.18.026704

[b12] Jing R. . Properties of Dy^3+^-doped Ge-As-Ga-Se Chalcogenide Glasses. J. Am. Ceram. Soc. 89, 2486–2491 (2006).

[b13] Heo J. & Yong B. S. Absorption and mid-infrared emission spectroscopy of Dy^3+^ in Ge-As(or Ga)-S glasses. J. Non-Cryst. Solids 196, 162–167 (1996).

[b14] Wei K., Machewirth D. P., Wenzel J., Snitzer E. & Sigel G. H. Spectroscopy of Dy^3+^ in Ge–Ga–S glass and its suitability for 1.3 μm fiber-optical amplifier applications. Opt. Lett. 19, 904–906 (1994). 19844483 10.1364/ol.19.000904

[b15] Lee T. H., Simdyankin S. I., Hegedus J., Heo J. & Elliott S. R. Spatial distribution of rare-earth ions and GaS_4_ tetrahedra in chalcogenide glasses studied via laser spectroscopy and ab initio molecular dynamics simulation. Phys. Rev. B 81, 760–762 (2010).

[b16] Ivanova Z. G., Vassilev V. S., Cernoskova E. & Cernosek Z. Physicochemical, structural and fluorescence properties of Er-doped Ge–S–Ga glasses. J. Phys. Chem. Solids 64, 107–110 (2003).

[b17] Neto J. A. M. . The application of Ga: La: S-based glass for optical amplification at 1.3 μm. J. Non-Cryst. Solids 184, 292–296 (1995).

[b18] Galstyan A., Messaddeq S. H., Segre C. U., Galstian T. & Messaddeq Y. Structural analysis of Tm^3+^ doped As–S–Ga glasses by Raman and EXAFS spectroscopy. J. Non-Cryst. Solids 432 (2015).

[b19] Tawarayama H. . Optical Amplification at 1.3 μm in a Praseodymium-Doped Sulfide-Glass Fiber. J. Am. Ceram. Soc. 83, 792–796 (2000).

[b20] Pethes I. . Local motifs in GeS_2_-Ga_2_S_3_ glasses. J. Alloy. Compd. 673, 149–157 (2016).

[b21] Yang A. . Ga-Sb-S chalcogenide glasses for mid-infrared applications. J. Am. Ceram. Soc. 99, 1–4 (2015).

[b22] Barnier S., Guittard M., Julien C. & Chilouet A. Study of the antimony environment in gallium-antimony-sulphur glasses -Phase diagram and infrared absorption investigations. Mater. Res. Bull. 28, 399–405 (1993).

[b23] Ichikawa M., Ishikawa Y. I., Wakasugi T. & Kadono K. Mid-infrared emissions from Ho^3+^ in Ga_2_S_3_-GeS_2_-Sb_2_S_3_ glass. J. Lumin. 132, 784–788 (2012).

[b24] Kaistha A., Modgil V. & Rangra V. S. Structural Characterization and Compositional Dependence of Optical Properties of Ge_16_Se_52_Te_32−*x*_, Sb_*x*_, (*x* = 0, 2, 4, 6, 8) Glassy Alloys. J. Electron. Mater. 44, 4747–4753 (2015).

[b25] Seddon A. B., Tang Z., Furniss D., Sujecki S. & Benson T. M. Progress in rare-earth-doped mid-infrared fiber lasers. Opt. Express 18, 26704–26719 (2010). 21165021 10.1364/OE.18.026704

[b26] Lin C. . Mechanism of the enhancement of mid-infrared emission from GeS_2_-Ga_2_S_3_ chalcogenide glass-ceramics doped with Tm^3+^. Appl. Phys. Lett. 100, 231910 (2012).

[b27] Heo J., Yoon J. M. & Ryou S. Y. Raman Spectroscopic Analysis on the Solubility Mechanism of La^3+^ in GeS_2_–Ga_2_S_3_ Glasses. J. Non-Cryst. Solids 238, 115–123 (1998).

[b28] Sen S., Ponader C. W. & Aitken B. G. Ge and As x-ray absorption fine structure spectroscopic study of homopolar bonding, chemical order, and topology in Ge-As-S chalcogenide glasses. Phys. Rev. B 64, 115–149 (2001).

[b29] Song J. H. . EXAFS investigation on the structural environment of Tm^3+^ in Ge–Ga–S–CsBr glasses. J. Non-Cryst. Solids 353, 1251–1254 (2007).

[b30] Kolimiets B., Goryunova N. & Shilo V. Glassy State in Chalcogenides. 456–460 (Publishing House of the USSR Academy of Sciences, Petersburg, 1960).

[b31] Higuchi H., Kanno R., Kawamoto Y., Takahashi M. & Kadono K. Local structures of Er^3+^ containing Ga_2_S_3_–GeS_2_–La_2_S_3_ glass. Phys. Chem. Glasses 40, 122–125 (1999).

[b32] Wang X. . Influence of La/Al ratio on the structure and spectroscopy of Tm^3+^ doped Al_2_O_3_ - La_2_O_3_ - SiO_2_ glasses. J. Alloy. Compd. 690, 583–588 (2017)

[b33] Yong G. C., Song J. H., Yong B. S. & Heo J. Chemical characteristics of Dy–S bonds in Ge–As–S glass. J. Non-Cryst. Solids 353, 1665–1669 (2007).

[b34] Marchese D. & Jha A. The structural aspects of the solubility of Pr^3+^ ions in GeS_2_-based glasses. J. Non-Cryst. Solids 213, 381–387 (1997).

[b35] Aitken B. G., Ponader C. W. & Quimby R. S. Clustering of rare earths in GeAs sulfide glass. C. R. Chim. 5, 865–872 (2002).

[b36] Pangavhane S. D. . Laser desorption ionization time-of-flight mass spectrometry of erbium-doped Ga-Ge-Sb-S glasses. Rapid Commun. Mass. Spectrom. 28, 1221–1232 (2014). 24760563 10.1002/rcm.6896

[b37] Zhang M. J. . Dy^3+^-doped Ga–Sb–S chalcogenide glasses for mid-infrared lasers. Mater. Res. Bull. 70, 55–59 (2015).

[b38] Moizan V. . Er^3+^-doped GeGaSbS glasses for mid-infrared fiber laser application: synthesis and rare earth spectroscopy. Opt. Mater. 31, 39–46 (2008).

[b39] Nakamoto K. Infrared and Raman Spectra of Inorganic and Coordination Compounds Part A: Theory and Applications in Inorganic Chemistry (ed. Nakamoto K.) 192–204 (Wiley, 2009).

[b40] Lucovsky G., Deneufville J. P. & Galeener F. L. Study of the optic modes of Ge_0.3_S_0.7_ glass by infrared and Raman spectroscopy. Phys. Rev. B 9, 1591–1597 (1974).

[b41] Ichikawa M., Wakasugi T. & Kadono K. Glass formation, physico-chemical properties, and structure of glasses based on Ga_2_S_3_–GeS_2_–Sb_2_S_3_ system. J. Non-Cryst. Solids 356, 2235–2240 (2010).

[b42] Julien C. . Raman and Infrared Spectroscopic Studies of Ge–Ga–Ag Sulphide Glasses. Mater. Sci. Eng. B 22, 191–200 (1994).

[b43] Musgraves J. D., Wachtel P., Gleason B. & Richardson K. Raman spectroscopic analysis of the Ge–As–S chalcogenide glass-forming system. J. Non-Cryst. Solids 386, 61–66 (2014).

[b44] Lucovsky G. & Martin R. M. A molecular model for the vibrational modes in chalcogenide glasses. J. Non-Cryst. Solids 8–10, 185–190 (1972).

